# Risk factors for excess deaths during lockdown among older users of secondary care mental health services without confirmed COVID‐19: A retrospective cohort study

**DOI:** 10.1002/gps.5610

**Published:** 2021-08-19

**Authors:** Shanquan Chen, Peter B. Jones, Benjamin R. Underwood, Emilio Fernandez‐Egea, Pei Qin, Jonathan R. Lewis, Rudolf N. Cardinal

**Affiliations:** ^1^ Department of Psychiatry University of Cambridge Cambridge UK; ^2^ NIHR Applied Research Collaboration East of England UK; ^3^ Cambridgeshire and Peterborough NHS Foundation Trust Fulbourn UK; ^4^ Department of Biostatistics and Epidemiology Shenzhen University Health Science Centre Shenzhen China

**Keywords:** COVID‐19, excess deaths, lockdown, retrospective cohort study, risk factors

## Abstract

**Objective:**

To investigate factors contributing to excess deaths of older patients during the initial 2020 lockdown beyond those attributable to confirmed COVID‐19.

**Methods:**

Retrospective cohort study comparing patients treated between 23 March 2020 and 14 June 2020, deemed exposed to the pandemic/lockdown, to patients treated between 18 December 2019 and 10 March 2020, deemed to be unexposed. Data came from electronic clinical records from secondary care mental health services in Cambridgeshire and Peterborough NHS Foundation Trust (CPFT), UK (catchment area population ∼0.86 million). Eligible patients were aged 65 years or over at baseline with at least 14 days' follow‐up, excluding patients diagnosed with confirmed or suspected SARS‐CoV‐2 infection. The primary outcome was all‐cause mortality.

**Findings:**

In the two cohorts, 3,073 subjects were exposed to lockdown and 4,372 subjects were unexposed; the cohorts were followed up for an average of 74 and 78 days, respectively. After controlling for confounding by sociodemographic factors, smoking status, mental comorbidities, and physical comorbidities, patients with dementia suffered an additional 53% risk of death (HR = 1.53, 95% CI = 1.02–2.31), and patients with severe mental illness suffered an additional 123% risk of death (HR = 2.23, 95% CI = 1.42–3.49). No significant additional mortality risks were identified from physical comorbidities, potentially due to low statistical power in that respect.

**Conclusion:**

During lockdown people with dementia or severe mental illness had a higher risk of death without confirmed COVID‐19. These data could inform future health service responses and policymaking to help prevent avoidable excess death during future outbreaks of this or a similar infectious disease.

## Key points

- Previous studies have explored the risk factors for death following COVID‐19 infection, but data regarding the totality of excess deaths is sparse
- We conducted a retrospective cohort study based on a large clinical record database, making a comparison with a historical control group
- We found that older people with dementia or serious mental illness had a higher risk of death without confirmed COVID‐19 under the circumstance of lockdown
- This study could have an impact on existing health systems and clinical practice in response to the excess death associated with COVID‐19 lockdown, and inform preparation for possible future outbreaks

## 1 BACKGROUND

The novel coronavirus disease COVID‐19 was declared a pandemic by the World Health Organization on 11 March 2020.^1^ To minimize the spread of COVID‐19, social isolation or social distancing (as part of more general measures designed to cut infection, collectively referred to as ‘lockdown’) have been implemented globally. The United Kingdom (UK) implemented ‘social distancing’ from 16 March 2020 and more stringent measures, ‘lockdown’, from 23 March 2020. During the lockdown, day‐to‐day contacts were reduced by requiring people to stay at home, closing most businesses and venues, and stopping all gatherings of more than two people in public.^2^ Increasing evidence indicates that this has been an effective prevention and control measure.^3^ However, besides the deaths directly attributable to COVID‐19, a large number of extra indirect deaths have occurred during lockdown.^4^, ^5^, ^6^ For example, in England and Wales, in addition to the 45,511 deaths associated with COVID‐19 infection from 14 Mar 2020 to 29 May 2020, a further 12,522 excess deaths have been reported.^6^ More detailed information about these excess deaths is sparse, making it difficult to design strategies to ameliorate them. We hypothesized that both physical diseases and mental disorders would contribute.

We conducted a retrospective cohort study to identify which previously documented factors were associated with excess death during the lockdown, beyond those attributed to confirmed COVID‐19. We focused on patients over the age of 65 as they represent the most vulnerable population for death.^5^, ^6^

## 2 MATERIALS AND METHODS

### 2.1 Study design and participants

We performed a retrospective cohort study using data from the electronic clinical records of secondary care mental health services of Cambridgeshire and Peterborough NHS Foundation Trust (CPFT), which provides mental health and community physical health services to a population of approximately 0.86 million people (18% of whom are aged 65 years or over) in the UK. The present study focused on patients in receipt of secondary care mental health services. CPFT's electronic clinical records contain patient information recorded during routine treatment, such as sociodemographic information, smoking status, diagnosis, prescription data in free text, and death status. Clinicians enter aspects of this information in a systematic and structured/standard way to ensure its accuracy.^7^

For the cohort of patients exposed to lockdown, data collection began on 23 March 2020, and we used available data up to 14 June 2020. Data representing an unexposed cohort were collected from 18 December 2019 to 10 March 2020. We took 10 March as the cut‐off date as there was already a widespread concern with regards to coronavirus in the period immediately prior to lockdown, which may already have been changing behaviour. In order to keep the follow‐up duration consistent with the exposed cohort, the starting point for the data collection of the unexposed cohort was 18 December 2019. Eligible patients were those aged 65 years or over at baseline and had at least two weeks' (14 days') follow‐up. The origin time for each patient was the start of data collection or their date of registration with CPFT secondary care mental health services, whichever was latest. Follow‐up was until the patients' final CPFT data was recorded, the date of their death, or the study end date, whichever occurred first. For the exposed cohort data, we excluded patients who were diagnosed with confirmed or suspected SARS‐CoV‐2 infection following the England official criteria.^8^

This study focuses on risk factors for deaths occurring after lockdown among older adults known to secondary care mental health services who did not have confirmed COVID‐19. It is impossible to classify an individual death (where not directly attributed to COVID‐19) as ‘excess’ or ‘expected’, but we analyse risk factors contributing disproportionately to death after lockdown (more so than before lockdown) via a statistical comparison with historical control periods, described below.

### 2.2 Data collection

Data were extracted via the CPFT Research database (NHS Research Ethics 17/EE/0442), specifically from a de‐identified copy of data from CPFT's Servelec RiO electronic clinical records system, which covers all CPFT secondary mental health services. The database operates on an opt‐out basis, so has data on nearly all such CPFT patients (at present totalling ∼208,000 across all ages). This database includes sociodemographic variables, smoking status, death, and documented morbidities (including mental disorders and physical diseases). We examined the following sociodemographic variables: age at baseline (years), gender (male vs. female), marital status (married, cohabiting or civil partnership vs. not), and ethnicity (White vs. others). Smoking status was defined as having been a current or past smoker. Death was ascertained by weekly linkage to national NHS Spine mortality data for all patients known to CPFT's RiO system.

Comorbidities were judged based on World Health Organization (WHO) International Statistical Classification of Diseases (ICD‐10) diagnoses and on prescription information. Diagnoses were presented in coded data. Medicine information was extracted from free text using GATE‐based natural language processing (NLP) software.^9^ The mental disorders we focused on included dementia (recorded with ICD‐10 codes F00‐F03 and G30, or taking cholinesterase inhibitors or memantine), substance misuse (F10‐F19), severe/serious mental illness (SMI) (F20‐F29, F30, and F31, or taking antipsychotics, accepting that some antipsychotics could be prescribed for other mental disorders), depression (F32 or F33, or taking antidepressants), anxiety (F41 or F42), reaction to severe stress (F43), eating disorders (F50), personality disorders (F60‐F69), intellectual disability (F70‐F79), and intentional self‐harm (X60‐X84). The physical diseases we focused on included diabetes mellitus (E10‐E14, or taking hypoglycaemic agents), circulation system diseases (including hypertension or cardiovascular or cerebrovascular disease, ICD‐10 codes I10‐I13, I15, I21‐I25, and I60‐I69, or taking ACE inhibitors, angiotensin‐II receptor antagonists, beta blockers, calcium channel antagonists, or diuretics), dyslipidemia (E78, or taking lipid‐lowering medications), respiratory diseases (including asthma or chronic obstructive pulmonary disease [COPD], indicated by ICD‐10 codes J44 and J45, or taking oral or inhaled corticosteroids, bronchodilators, or anti‐inflammatory drugs used for airways disease, accepting that oral corticosteroids may also indicate other inflammatory disorders), and cancer (C00‐C97, or taking drugs implying cancer). Identification of these diseases was based on historical CPFT records up to one year before the origin time of follow up, except for lifelong diseases including dementia, SMI, and the aforementioned physical diseases. The medicines referred to were selected according to UK National Institute for Health and Care Excellence (NICE) guidelines. See Table S1.

Data were complete for the outcome and for predictors, except for ethnicity, and if diagnoses/medications were under‐coded (the degree of undercoding not being directly measurable in this data set). We omitted cases because of missing ethnicity, rather than attempting to impute a value.

### 2.3 Statistical analysis

To describe the baseline characterizes of this cohort, category variables were reported as number (percentage), and continuous variables were reported as mean (standard deviation).

Cox proportional hazard models were used to predict death by risk factors. Risk factors such as sociodemographic variables, smoking status, mental disorders, and physical diseases were treated as time‐independent variables. A binary variable (exposed vs. unexposed) was treated as a time‐dependent variable by controlling it as a stratification factor in the Cox model. To examine whether associations between risk factors and death changed under lockdown, interactions between risk factors and this stratification factor were included in the Cox model. Such an interaction, for a given risk factor, judges whether its contribution to the risk of death differed before versus after lockdown. The two cohorts were collected from the same database (CPFT) but at different time periods. Participants in the unexposed cohort would also be participants in the exposed cohort, if they survived until 23 March 2020 (the start of the exposed cohort period). We considered this overlap by performing the Cox regression clustered on patients.

A full model with all risk factors and all interactions was fitted first, then a stepwise process based on the Akaike information criterion (AIC) was conducted. The model with the lowest AIC was chosen as the final model.

We performed three sensitivity analyses. First, we required a longer follow‐up (at least three weeks, 21 days) to exclude patients registered in CPFT temporarily, such as visitors or people discharged after a single assessment. Second, to control for a seasonal change effect on the death toll, we also analysed data from the same period of the previous two years, namely between 23 March 2019 and 14 June 2019 and between 23 March 2018 and 14 June 2018, as an alternative unexposed group. Third, given the level of missing ethnicity data, we repeated the analysis without ethnicity as a covariate, including those who had been excluded before.

Multicollinearity was tested using the variance inflation factor (VIF). VIF ≥10 indicates a sign of severe or serious multicollinearity.^10^ In this study, all models had a maximum VIF of 2.3, suggesting a negligible amount of multicollinearity.

We used R (version 3.5.0) for all analyses and defined statistical significance as *p* < 0.05. Results are reported following the STROBE checklist for cohort studies.^11^

### 2.4 Role of the funding source

The funder of the study had no role in study design, data collection, data analysis, data interpretation or writing of the article. SC, JL, and RNC had full access to all the data in the study. The corresponding author had final responsibility for the decision to submit for publication.

## 3 RESULTS

**FIGURE 1:**
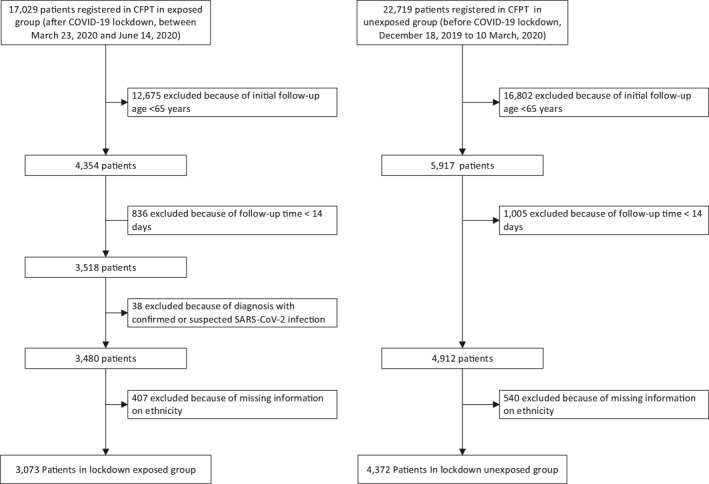
STROBE diagram showing construction of the cohorts

After omitting cases because of missing ethnicity information (omitted cases accounted for 11.7% in the lockdown‐exposed group and 11.0% in the lockdown‐unexposed group), we followed 3,073 patients exposed to COVID‐19 lockdown during the pandemic and 4,372 patients before the COVID‐19 lockdown (unexposed group) (Figure 1). The cohorts were followed up for an average of 74 and 78 days, respectively. In the cohort exposed to lockdown, there were 197 (6.4%) deaths that were not directly attributed to COVID‐19, a significantly higher fraction (*p* = 0.0001) than in the unexposed cohort (187%, 4.3%).

There were some minor differences in the baseline characteristics of patients between the two cohorts (Table 1). Overall, patients in the exposed cohort were slightly younger (mean age: 78.89 vs. 79.44 years), had less comorbid dementia (41.2% vs. 45.7%), had more comorbid SMI (34.5% vs. 30%), and had more comorbid depression (51% vs. 48%). No significant difference between the two groups was observed for other characteristics, including gender, marital status, ethnicity, smoking status, and other mental or physical comorbidities.

**TABLE 1:** Patient characteristics

Variable	Exposed group (*n* = 3,073)	Unexposed group (*n* = 4,372)	Test statistic	*p*
Age (years)	78.89 (7.94)	79.44 (7.99)	*t* = 2.929	**0.0034**
Gender (= male)	1277 (41.6%)	1824 (41.7%)	*χ* ^2^ = 0.014	0.9061
Marital status (= married, cohabiting or civil partnership)	1251 (40.7%)	1749 (40%)	*χ* ^2^ = 0.344	0.5576
Ethnicity (= White)	2908 (94.6%)	4142 (94.7%)	*χ* ^2^ = 0.024	0.8782
Smoker (= current or former)	83 (2.7%)	100 (2.3%)	*χ* ^2^ = 1.121	0.2897
Mental disorders				
Dementia (= true)	1267 (41.2%)	1997 (45.7%)	*χ* ^2^ = 14.315	**0.0002**
Substance misuse (= true)	29 (0.9%)	33 (0.8%)	*χ* ^2^ = 0.568	0.4511
Severe mental illness (= true)	1059 (34.5%)	1312 (30%)	*χ* ^2^ = 16.277	**0.0001**
Depression (= true)	1567 (51%)	2097 (48%)	*χ* ^2^ = 6.500	**0.0108**
Anxiety (= true)	128 (4.2%)	171 (3.9%)	*χ* ^2^ = 0.240	0.6243
Reaction to severe stress (= true)	86 (2.8%)	103 (2.4%)	*χ* ^2^ = 1.256	0.2624
Eating disorder (= true)	4 (0.1%)	4 (0.1%)	*χ* ^2^ = 0.020	0.8869
Personality disorder (= true)	31 (1%)	36 (0.8%)	*χ* ^2^ = 0.503	0.4782
Intellectual disability (= true)	8 (0.3%)	9 (0.2%)	*χ* ^2^ = 0.057	0.8117
Intentional self‐harm (= true)	18 (0.6%)	23 (0.5%)	*χ* ^2^ = 0.034	0.8544
Physical diseases				
Diabetes mellitus (= true)	496 (16.1%)	701 (16%)	*χ* ^2^ = 0.008	0.9272
Cardiovascular diseases (= true)	2173 (70.7%)	3097 (70.8%)	*χ* ^2^ = 0.008	0.9280
Cancer (= true)	52 (1.7%)	68 (1.6%)	*χ* ^2^ = 0.135	0.7129
Dyslipidemia (= true)	1436 (46.7%)	2047 (46.8%)	*χ* ^2^ = 0.003	0.9570
Respiratory diseases (= true)	470 (15.3%)	659 (15.1%)	*χ* ^2^ = 0.053	0.8186
Death (= true)	197 (6.4%)	187 (4.3%)	*χ* ^2^ = 16.358	**0.0001**
Follow‐up duration(days)	74.47 (20.07)	78.02 (16.11)	*t* = 8.123	**< 0.0001**

*Note*: Data are shown as mean (standard deviation) or number (percentage). *P* values for age and time‐to‐event were obtained by *t* test, for eating disorders via Fisher's exact test, and for others via Pearson's chi‐square test. Bold print in the final column indicates *p* < 0.05.

**FIGURE 2:**
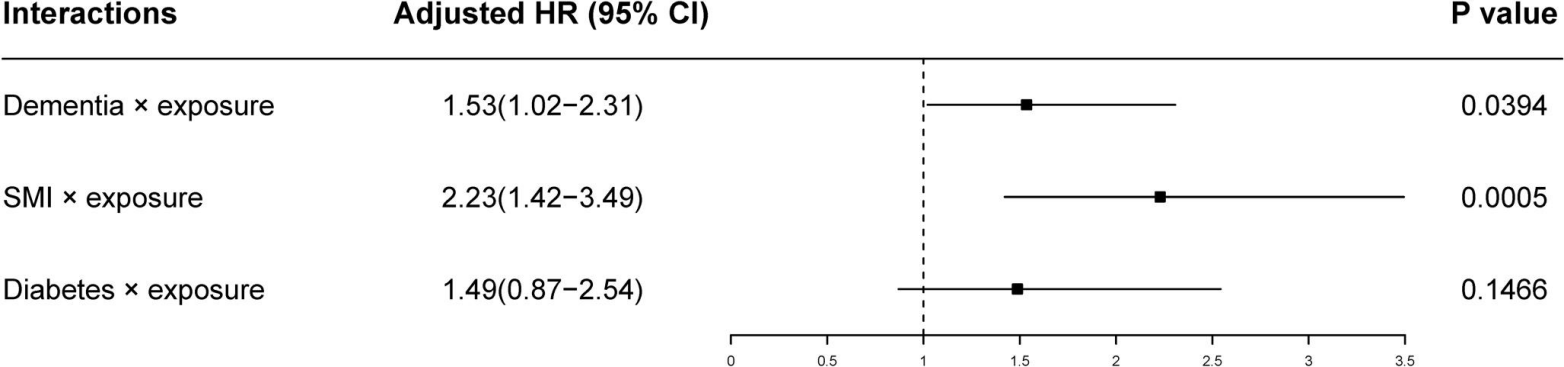
Adjusted hazard ratios (HR) for excess death during lockdown. Data are fitted by the Cox model. A binary variable (exposed vs. unexposed) was treated as a time‐dependent variable and as a stratification factor in the Cox model. Risk factors' extra effects on death during lockdown were tested via the interactions between risk factors and exposure. After model selection based on the Akaike information criterion (AIC), the final predictive factors included in the Cox model were age, gender, marital status, ethnicity, dementia, serious/severe mental illness (SMI), anxiety, diabetes, circulatory system diseases, exposure, dementia × exposure, SMI × exposure, and diabetes × exposure. Only the results of the interactions are shown

After controlling for confounding by sociodemographic factors, smoking status, other mental comorbidities, and physical comorbidities, patients with dementia in the lockdown‐exposed group suffered an additional 53% risk of death (HR = 1.53, 95% CI = 1.02‐2.31), and patients with SMI suffered an additional 123% risk of death (HR = 2.23, 95% CI = 1.42–3.49) (Figure 2). No significant additional death risks were identified from physical comorbidities.

Sensitivity analyses using a longer follow‐up (Figure S1), to exclude a seasonal effect (Figure S2), or considering people with missing ethnicity data (Figure S3) all confirmed our primary results.

## 4 DISCUSSION

### 4.1 Principal findings and strengths

This study is the first to investigate risk factors contributing to excess death among older patients of a mental health NHS Trust during the lockdown. Using a retrospective cohort study based on a large and comprehensive clinical record database, with comparison to a control group, we found that older people with dementia or SMI had a higher risk of excess death during lockdown. This is in contrast to previous findings^12^, ^13^, ^14^, ^15^, ^16^, ^17^ that physical comorbidities were the main risk factors for death from COVID‐19 patients among the elderly. Unexpectedly, physical diseases in this study were not identified as risk factors for the excess death for those patients without confirmed COVID‐19 under the circumstance of lockdown (likely due to relatively low power in that respect, as discussed below).

### 4.2 Interpretation

Several other studies^18^, ^19^ have focused on excess deaths, and the US Centers for Disease Control and Prevention (CDC) have developed a method,^20^ based on the overdispersed Poisson distribution, to calculate the number of excess deaths. By itself, this approach provides information on the number of excess deaths. Our study attempted to go beyond this by focusing on vulnerable populations and asking which specific factors were associated with excess death, not directly attributed to COVID‐19 (also by statistical comparison to a historical control period). Such questions may be important in practice to guide targeted policy formulation and resource allocation.

Our results indicate that older people with dementia suffered an increased likelihood of death during lockdown. Generally, individuals with dementia are more likely to have chronic physical diseases^21^ such as diabetes and cardiovascular disease, and are more likely to depend on family or caregivers as compared with their counterparts without dementia.^22^ Despite controlling for these other known risk factors for mortality from COVID‐19, dementia remained an independent risk factor for excess death. This finding is to some extent in keeping with another UK study suggesting that dementia is a relatively strong risk factor for death from COVID‐19 in hospital (HR = 1.79, 95% CI 1.67–1.93).^23^ One possibility is that these deaths are due to unrecognized infection with SARS‐CoV‐2. This may not be reflected in the recorded cause of death either, because the patient is near the end of life with dementia (in which case dementia may be recorded) or because the frail elderly have an atypical presentation of infection.^24^, ^25^ Many such patients stop eating and die relatively rapidly without developing characteristic fever or cough, though pathological testing has subsequently revealed infection with SARS‐CoV‐2.^24^, ^25^ Alternatively, the excess mortality may be due to indirect effects of the coronavirus outbreak and not direct infection with the virus. The lockdown may make daily support less available, and therefore expose people to risks such as discontinuation of medication.^22^ During lockdown, social support has been reduced, which may have increased loneliness and abandonment, perhaps particularly for patients unable to understand the reasons for this, and this may impact mortality.^26^, ^27^, ^28^ All of these could result in a worsening of neuropsychiatric symptoms. Indeed, concerns have been expressed that antipsychotic prescribing may increase to manage behavioural disturbance in dementia during the COVID‐19 pandemic,^29^ and antipsychotics increase mortality.^30^ It is also possible that these patients did not seek or receive the normal standard of care for their physical co‐morbidities. Documented physical comorbidities were not closely associated with mortality in this study, but the possibility of undercoding of physical comorbidities in secondary care mental health services remains (discussed further below), and in that respect, and in terms of total sample size, our study may have been underpowered to detect the effect of physical comorbidities, which have been established in large‐scale population studies.^23^

Our findings also indicated that older people with SMI also suffered an increased likelihood of death during lockdown. For people with SMI, the lockdown could further reduce and collapse potentially already tenuous social networks,^31^, ^32^ potentially exacerbating feelings of perplexity, anxiety, and paranoia.^31^, ^33^ The lockdown could also disrupt continuity of care,^32^ which is critical to prevent decompensation and its consequences, such as mental and physical deterioration, and even death.^34^ In addition, lockdown could reduce patients' access to effective treatment;^35^ for example, optimal care for schizophrenia includes assertive community treatment and intensive case management, which emphasize in‐person contacts.^36^ Telehealth might not add much value to care as usual.^37^ As discussed above, a reduction in health‐seeking for new‐onset physical disorders may have been contributory in this group.

For both group of people with dementia or SMI, we cannot exclude the possibility that lockdown increased their suicide rates, as death certificate causes of death were not available (see below); further evidence is required, but there has been no evidence for a rise in suicide in England following lockdown.^38^, ^39^

### 4.3 Limitations

Our study's limitations include the following.

#### 4.3.1 Causes of death

We did not have data on causes of death (from the UK death certification process), and therefore could not study the detailed reasons for excess death (not associated with documented COVID‐19 infection) in patients with dementia or SMI. This limitation needs attention in future studies.

#### 4.3.2 Under‐recognition of COVID‐19

Patients with dementia or SMI may be groups with difficulties in recognizing and reporting COVID‐19 symptoms. As discussed above, death might be recorded but not mention coronavirus, even though it might be a coronavirus‐related death. We have tried to reduce this possibility by excluding patients with suspected as well as confirmed COVID‐19.

#### 4.3.3 Under‐recording of COVID‐19

Although it is highly likely that all patients who were tested by CPFT had their COVID‐19 status recorded, it is possible that patients were diagnosed with COVID‐19 elsewhere and this was not reflected in their CPFT records. We only included people with at least 14 days' follow‐up, reducing the chance that people later diagnosed with COVID‐19 were included. However, it is still possible that not all such patients were excluded.

#### 4.3.4 Implications of a mental health service cohort

CPFT is a mental health and community NHS trust, providing both physical and mental health services, and in this study we examined data from patients over 65 known to secondary care mental health services. An advantage is that CPFT sees a relatively high proportion of those with severe mental illness. However, the low prevalence of certain mental disorders (e.g. substance use, anxiety, reactions to severe stress, and eating disorders), which may be due to secondary care bias or undercoding, may have reduced power to detect an effect of these conditions. A disadvantage is that physical disorders may have been undercoded. We attempted to mitigate this by using data derived from free text (as well as coded diagnoses) to detect physical disorders, and by using up to a year's records to identify physical diseases. However, the potential for undercoding may have reduced power in this context.

#### 4.3.5 Community versus inpatient status

Our data included patients in the community (outpatients) and inpatients, who may have had different vulnerabilities. However, the psychiatric inpatient proportion was very small, so the present study was primarily of a outpatient cohort; see Chen et al. (2020)^40^ for a description of psychiatric inpatient numbers across all ages during this period. We did not have data on acute hospital admissions for our cohorts.

#### 4.3.6 Residential setting

We were unable to control for patients' residential setting, since our data did not include this. People with dementia are more likely to be in care homes than those with SMI. People in living in semi‐shared accommodation (such as care homes) may have a higher probability of cross‐infection. We attempted to mitigate this by excluding people with suspected as well as confirmed COVID‐19, but the influence of missing information about residential setting, be it under‐ or overestimation, is unknown.

#### 4.3.7 Severity of comorbidities

The impact of severe diseases (e.g., advanced cancer, severe dementia, uncontrolled diabetes) may be different from that exerted by milder clinical conditions, but information was not available on the severity of the chronic conditions we analysed.

#### 4.3.8 Winter comparator cohort

Using winter data as our “unexposed” group could have led to underestimation of excess deaths, because more deaths occur among the elderly during winter months (Dec‐Mar) than in other seasons.^41^ Our sensitivity analysis on seasonal effects to some extent confirmed this: both dementia and SMI remained significant risk factors for death (Figure S2), but the size of this effect in dementia was greatly increased, and for SMI it was reduced.

#### 4.3.9 Potential for unrecognized early COVID‐19

The first COVID‐19 case was confirmed in the UK on 31 January 2020, and population behaviour may have changed even before the lockdown; similarly, five COVID‐19 cases were recognized in our region up to 10 March, but it is certainly likely that some early cases went unrecognized, so some people with COVID‐19 may have been in the “unexposed” cohort. However, such an effect would tend to reduce power (by increasing mortality in the control cohort) rather than produce a Type I error; likewise, the sensitivity analyses support the consistency of our results.

#### 4.3.10 Cohort overlap

We analysed patients with an active case record and not solely new referrals, and thus some patients may have been present in both cohorts (particularly those receiving long‐term community care). As discussed in the Methods, we considered this overlap via regression clustered on patients.

#### 4.3.11 Generalizability

Our findings may not generalize. However, measures such as social distancing and lockdown are being adopted in similar ways internationally, so the results of the present study may be useful for other regions and countries.

## 5 CONCLUSION

We report some of the factors associated with excess death in the context of the COVID‐19 pandemic and the social measures to prevent its spread. Dementia and SMI were identified as two major risk factors for excess mortality in patients over 65 in receipt of mental health services. We suggest this information could be used to focus attention and resource on patients with dementia or SMI in such circumstances. This should be used to support clinical guidelines and policy at all levels during this outbreak and for any future events.

## CONFLICT OF INTERESTS

Shanquan Chen, Emilio Fernandez‐Egea, Pei Qin, and Jonathan R. Lewis declare no conflict of interest with this work. Peter B. Jones is a scientific advisory board member for Janssen and Recordati. Benjamin R. Underwood was clinical director for older people's and adult community services at CPFT during much of the pandemic. He is clinical director of the Windsor Unit at Fulbourn Hospital (CPFT), which delivers clinical trials in dementia/mild cognitive impairment for academic and commercial organisations without personal benefit, and is the clinical lead for dementia for the NIHR Clinical Research Network (CRN) in the East of England. His salary is part‐funded by the NIHR CRN. He has been principal investigator on trials for Axovant, Lilly, and EIP Pharma; his institution has benefited from payment for research carried out but he has not personally received any money. His lectureship is funded by Gnodde Goldman‐Sachs Giving. His wife is the lead for mental health for Suffolk Clinical Commissioning Group. Rudolf N. Cardinal consults for Campden Instruments Ltd and receives royalties from Cambridge University Press, Cambridge Enterprise, and Routledge.

## ETHICAL STATEMENT

NHS Research Ethics 17/EE/0442.

## AUTHOR CONTRIBUTIONS

Shanquan Chen and Rudolf N. Cardinal contributed to the study design. Shanquan Chen and Jonathan R. Lewis processed the data. Shanquan Chen conducted the statistical analyses. Shanquan Chen wrote the first draft. All authors edited and approved the final manuscript.

## PATIENT AND PUBLIC INVOLVEMENT

No patients were involved in the development of the research question or the outcome measures, or in developing plans for the design and analysis of the study.

## Supporting information

Supporting Information S1

## Data Availability

Patient‐level data are not publicly available, under NHS Research Ethics terms. Source code and summary data are available on request. Data sharing is not applicable to this article as no new data were created or analysed in this study.

## References

[1] World Health Organization . Timeline of WHO's Response to COVID‐19; 2020. https://www.who.int/news‐room/detail/29‐06‐2020‐covidtimeline. Accessed July 7, 2020. Published.

[2] Cabinet Office UK . Staying at Home and Away from Others (Social Distancing). https://www.gov.uk/government/publications/full‐guidance‐on‐staying‐at‐home‐and‐away‐from‐others March 23, 2020. Accessed August 3, 2020. Published.

[3] Chu DK , Akl EA , Duda S , et al. Physical distancing, face masks, and eye protection to prevent person‐to‐person transmission of SARS‐CoV‐2 and COVID‐19: a systematic review and meta‐analysis. Lancet. 2020;395(10242):1973‐1987. 32497510 10.1016/S0140-6736(20)31142-9 PMC7263814

[4] Piccininni M , Rohmann JL , Foresti L , Lurani C , Kurth T . Use of all cause mortality to quantify the consequences of covid‐19 in Nembro, Lombardy: descriptive study. BMJ. 2020;369:m1835. 32409488 10.1136/bmj.m1835 PMC7223479

[5] Magnani C , Azzolina D , Gallo E , Ferrante D , Gregori D . How large was the mortality increase directly and indirectly caused by the COVID‐19 epidemic? An analysis on all‐causes mortality data in Italy. Int J Environ Res Public Health. 2020;17(10). 10.3390/ijerph17103452 PMC7277828 32429172

[6] Office for National Statistics . Deaths Registered Weekly in England and Wales; 2020. provisional: week ending 29 May 2020. https://www.ons.gov.uk/peoplepopulationandcommunity/birthsdeathsandmarriages/deaths/bulletins/deathsregisteredweeklyinenglandandwalesprovisional/latest. Accessed September 6, 2020. Published.

[7] Price A , Farooq R , Yuan JM , Menon VB , Cardinal RN , O'Brien JT . Mortality in dementia with Lewy bodies compared with Alzheimer's dementia: a retrospective naturalistic cohort study. BMJ Open. 2017;7(11):e017504. 10.1136/bmjopen-2017-017504 PMC5695389 29101136

[8] Public Health England COVID‐19: investigation and initial clinical management of possible cases. 2020. https://www.gov.uk/government/publications/wuhan‐novel‐coronavirus‐initial‐investigation‐of‐possible‐cases/investigation‐and‐initial‐clinical‐management‐of‐possible‐cases‐of‐wuhan‐novel‐coronavirus‐wn‐cov‐infection. Accessed July 30, 2020. Published.

[9] Cardinal RN . Clinical records anonymisation and text extraction (CRATE): an open‐source software system. BMC Med Inform Decis Mak. 2017;17(1):50. 28441940 10.1186/s12911-017-0437-1 PMC5405523

[10] O'Brien RM . A caution regarding rules of thumb for variance inflation factors. Qual Quant. 2007;41(5):673‐690.

[11] STROBE . STROBE Checklist for Cohort Studies; 2007. https://www.strobe‐statement.org/fileadmin/Strobe/uploads/checklists/STROBE_checklist_v4_cohort.pdf. Accessed July 30, 2020. Published.

[12] Du RH , Liang LR , Yang CQ , et al. Predictors of mortality for patients with COVID‐19 pneumonia caused by SARS‐CoV‐2: a prospective cohort study. Eur Respir J. 2020;55(5). 10.1183/13993003.00524-2020 PMC7144257 32269088

[13] Wang K , Zhang Z , Yu M , Tao Y , Xie M . 15‐day mortality and associated risk factors for hospitalized patients with COVID‐19 in Wuhan, China: an ambispective observational cohort study. Intensive Care Med. 2020;46(7):1472‐1474. 32328724 10.1007/s00134-020-06047-w PMC7176814

[14] Zhou F , Yu T , Du R , et al. Clinical course and risk factors for mortality of adult inpatients with COVID‐19 in Wuhan, China: a retrospective cohort study. Lancet. 2020;395(10229):1054‐1062. 32171076 10.1016/S0140-6736(20)30566-3 PMC7270627

[15] Yang K , Sheng Y , Huang C , et al. Clinical characteristics, outcomes, and risk factors for mortality in patients with cancer and COVID‐19 in Hubei, China: a multicentre, retrospective, cohort study. Lancet Oncol. 2020;21(7):904‐913. 32479787 10.1016/S1470-2045(20)30310-7 PMC7259917

[16] Lee LY , Cazier JB , Angelis V , et al. COVID‐19 mortality in patients with cancer on chemotherapy or other anticancer treatments: a prospective cohort study. Lancet. 2020;395(10241):1919‐1926. 32473682 10.1016/S0140-6736(20)31173-9 PMC7255715

[17] Shi Q , Zhang X , Jiang F , et al. Clinical characteristics and risk factors for mortality of COVID‐19 patients with diabetes in Wuhan, China: a two‐center, retrospective study. Diabetes Care. 2020;43(7):1382‐1391. 32409504 10.2337/dc20-0598

[18] Banerjee A , Pasea L , Harris S , et al. Estimating excess 1‐year mortality associated with the COVID‐19 pandemic according to underlying conditions and age: a population‐based cohort study. Lancet. 2020;395(10238):1715‐1725. 32405103 10.1016/S0140-6736(20)30854-0 PMC7217641

[19] Bone AE , Finucane AM , Leniz J , Higginson IJ , Sleeman KE . Changing patterns of mortality during the COVID‐19 pandemic: population‐based modelling to understand palliative care implications. Palliat Med. 2020;34(9):1193‐1201. 32706299 10.1177/0269216320944810 PMC7385436

[20] Centers for Disease Control and Prevention . Excess Deaths Associated with COVID‐19; 2020. https://www.cdc.gov/nchs/nvss/vsrr/covid19/excess_deaths.htm#references. Accessed September 15, 2020. Published.

[21] Bauer K , Schwarzkopf L , Graessel E , Holle R . A claims data‐based comparison of comorbidity in individuals with and without dementia. BMC Geriatr. 2014;14:10. 24472217 10.1186/1471-2318-14-10 PMC3909381

[22] Brown EE , Kumar S , Rajji TK , Pollock BG , Mulsant BH . Anticipating and mitigating the impact of the COVID‐19 pandemic on Alzheimer's disease and related dementias. Am J Geriatric Psychiatry. 2020;28(7):712‐721. 10.1016/j.jagp.2020.04.010 PMC7165101 32331845

[23] Williamson EJ , Walker AJ , Bhaskaran K , et al. Factors associated with COVID‐19‐related death using OpenSAFELY. Nature. 2020;584(7821):430–436. 32640463 10.1038/s41586-020-2521-4 PMC7611074

[24] Isaia G , Marinello R , Tibaldi V , Tamone C , Bo M . Atypical presentation of COVID‐19 in an older adult with severe Alzheimer disease. Am J Geriatric Psychiatry. 2020;28(7):790‐791. 10.1016/j.jagp.2020.04.018 PMC7175908 32381283

[25] Tay HS , Harwood R . Atypical presentation of COVID‐19 in a frail older person. Age Ageing. 2020;49(4):523‐524. 32315386 10.1093/ageing/afaa068 PMC7188159

[26] Wang H , Li T , Barbarino P , et al. Dementia care during COVID‐19. Lancet. 2020;395(10231):1190‐1191. 32240625 10.1016/S0140-6736(20)30755-8 PMC7146671

[27] Lara B , Carnes A , Dakterzada F , Benitez I , Pinol‐Ripoll G . Neuropsychiatric symptoms and quality of life in Spanish patients with Alzheimer's disease during the COVID‐19 lockdown. Eur J Neurol. 2020;27(9):1744‐1747. 32449791 10.1111/ene.14339 PMC7283827

[28] Goodman‐Casanova JM , Dura‐Perez E , Guzman‐Parra J , Cuesta‐Vargas A , Mayoral‐Cleries F . Telehealth home support during COVID‐19 confinement for community‐dwelling older adults with mild cognitive impairment or mild dementia: survey study. J Med Internet Res. 2020;22(5):e19434. 32401215 10.2196/19434 PMC7247465

[29] Velayudhan L , Aarsland D , Ballard C . Mental health of people living with dementia in care homes during COVID‐19 pandemic. Int. Psychogeriatr. 2020;32(10):1253‐1254. 32487278 10.1017/S1041610220001088 PMC7302947

[30] Yeh TC , Tzeng NS , Li JC , et al. Mortality risk of atypical antipsychotics for behavioral and psychological symptoms of dementia: a meta‐analysis, meta‐regression, and trial sequential analysis of randomized controlled trials. J Clin Psychopharmacol. 2019;39(5):472‐478. 31433335 10.1097/JCP.0000000000001083

[31] Shinn AK , Viron M . Perspectives on the COVID‐19 pandemic and individuals with serious mental illness. J Clin Psychiatry. 2020;81(3). 10.4088/JCP.20com13412 32369691

[32] Kozloff N , Mulsant BH , Stergiopoulos V , Voineskos AN . The COVID‐19 global pandemic: implications for people with schizophrenia and related disorders. Schizophr Bull. 2020;46(4):752‐757. 32343342 10.1093/schbul/sbaa051 PMC7197583

[33] Iasevoli F , Fornaro M , D'Urso G , et al. Psychological distress in patients with serious mental illness during the COVID‐19 outbreak and one‐month mass quarantine in Italy. Psychol Med. 2021;51(6):1054‐1056. https://pubmed.ncbi.nlm.nih.gov/32423496/ 32423496 10.1017/S0033291720001841 PMC7261960

[34] Anderson KK , Norman R , MacDougall A , et al. Effectiveness of early psychosis intervention: comparison of service users and nonusers in population‐based health administrative data. Am J Psychiatry. 2018;175(5):443‐452. 29495897 10.1176/appi.ajp.2017.17050480

[35] Nichols J , Gannon JM , Conlogue J , et al. Ensuring care for clozapine‐treated schizophrenia patients during the COVID‐19 pandemic. Schizophrenia Res. 2020;222:499‐500. 10.1016/j.schres.2020.05.053 PMC7247984 32473934

[36] Mueser KT , Bond GR , Drake RE , Resnick SG . Models of community care for severe mental illness: a review of research on case management. Schizophr Bull. 1998;24(1):37‐74. 9502546 10.1093/oxfordjournals.schbul.a033314

[37] Hulsbosch AM , Nugter MA , Tamis P , Kroon H . Videoconferencing in a mental health service in The Netherlands: a randomized controlled trial on patient satisfaction and clinical outcomes for outpatients with severe mental illness. J Telemedicine Telecare. 2017;23(5):513‐520. 10.1177/1357633X16650096 27236703

[38] Appleby L , Richards N , Ibrahim S , Turnbull P , Rodway C , Kapur N . Suicide in England in the COVID‐19 pandemic: early observational data from real time surveillance. Lancet Regional Health Europe. 2021;4:100110. 34557817 10.1016/j.lanepe.2021.100110 PMC8454726

[39] Appleby L . What has been the effect of covid‐19 on suicide rates? BMJ. 2021;372:n834. 33782026 10.1136/bmj.n834

[40] Chen S , Jones PB , Underwood BR , et al. The early impact of COVID‐19 on mental health and community physical health services and their patients' mortality in Cambridgeshire and Peterborough, UK. J Psychiatric Res. 2020;131:244‐254. 10.1016/j.jpsychires.2020.09.020 PMC7508053 33035957

[41] Kmietowicz Z . Excess winter deaths rose by 29% last year from 2011‐12. BMJ. 2013;347:f7093. 24284052 10.1136/bmj.f7093

