# Supplementary material for: Risk factors for excess deaths during lockdown among older users of secondary care mental health services without confirmed COVID‐19: A retrospective cohort study
Source: Int J Geriatr Psychiatry. 2021 Aug 19;36(12):1899–907. doi: 10.1002/gps.5610 (PMC8420159; doi:10.1002/gps.5610)

**Supplementary Table 1: List of medicines**

| Cholinesterase inhibitors and glutamate receptor antagonists | **Cholinesterase inhibitors:**  Donepezil, galantamine, rivastigmine  **Glutamate receptor antagonists:**  Memantine |
| --- | --- |
| Antipsychotics | **First-generation:** Benperidol, chlorpromazine, flupentixol, fluphenazine, haloperidol, levomepromazine, pericyazine, perphenazine, pimozide, pipotiazine, prochlorperazine, promazine, trifluoperazine, zuclopenthixol  **Second-generation:** Asenapine, amisulpride, aripiprazole, clozapine, iloperidone, lurasidone, olanzapine, paliperidone, quetiapine, risperidone, sertindole, sulpiride, ziprasidone, zotepine |
| Antidepressants | Agomelatine, amitriptyline, bupropion, citalopram, clomipramine, dosulepin, doxepin, duloxetine, escitalopram, fluoxetine, fluvoxamine, imipramine, isocarboxazid, lofepramine, maprotiline, mianserin, mirtazapine, moclobemide, nefazodone, nortriptyline, paroxetine, phenelzine, reboxetine, sertraline, tranylcypromine, trazodone, trimipramine, tryptophan, venlafaxine, vortioxetine |
| Hypoglycaemic agents | Acarbose, alogliptin, canagliflozin, dapagliflozin, empagliflozin, exenatide, glibenclamide, gliclazide, glimepiride, glipizide, insulin, linagliptin, liraglutide, lixisenatide, metformin, nateglinide, pioglitazone, repaglinide, saxagliptin, sitagliptin, tolbutamide, vildagliptin |
| ACE inhibitors | Captopril, enalapril, fosinopril, imidapril, lisinopril, perindopril, quinapril, ramipril, trandolapril |
| Angiotensin-II receptor antagonists | Azilsartan, candesartan, eprosartan, irbesartan, losartan, olmesartan, telmisartan, valsartan |
| Beta blockers | Acebutolol, atenolol, bisoprolol, carvedilol, celiprolol hydrochloride  Labetalol, metoprolol, nadolol, nebivolol, pindolol, sotalol |
| Calcium channel antagonists | Amlodipine, diltiazem, elodipine, felodipine, lacidipine, lercanidipine, nicardipine, nifedipine, nimodipine, verapamil |
| Diuretics | **Thiazides:**  Bendroflumethiazide, chlortalidone, indapamide, metolazone, xipamide  **Loop diuretics:**  Bumetanide, furosemide, torasemide  **Potassium-sparing diuretics:**  Amiloride, triamterene  **Aldosterone antagonists:**  Eplerenone, spironolactone |
| Lipid-lowering medication | **Statins:**  Atorvastatin, fluvastatin, pravastatin, rosuvastatin, simvastatin  **Others:**  Colestyramine, colestipol, ezetimibe, fenofibrate |
| Corticosteroids, bronchodilators and other anti-inflammatory drugs for airways disease | **Corticosteroids:**  Alclometasone, betamethasone, clobetasol, clobetasone butyrate, fluocortolone, dexamethasone, fludrocortisone, fludroxycortide, flumetasone, fluocinolone, fluocinonide, fluorometholone, hydrocortisone, loteprednol, methylprednisolone, mometasone, prednisolone, triamcinolone  **Inhaled corticosteroids:**  Beclomethasone, budesonide, ciclesonide, fluticasone  **Bronchodilators and anti-inflammatory drugs used for airways disease:**  Aminophylline, formoterol, ipratropium, mepolizumab, montelukast, nedocromil sodium, omalizumab, reslizumab, salbutamol, salmeterol, sodium cromoglicate, terbutaline, theophylline, tiotropium |
| Drugs implying cancer | Docetaxel, clodronate, raloxifene, anastrozole, paclitaxel, pertuzumab, tamoxifen, trastuzumab, abiraterone acetate, bicalutamide, denosumab, enzalutamide |

**Supplementary Figure 1: Hazard ratios (HR) for excess death during lockdown, sensitivity analysis by requiring longer follow-up.** Data are fitted by the Cox model. A binary variable (exposed v. unexposed) was treated as a time-dependent variable and as a stratification factor in the Cox model. Risk factors’ extra effects on death during lockdown were tested via the interactions between risk factors and exposure. After model selection based on Akaike’s Information Criterion (AIC), the final predictive factors included in the Cox model were age, gender, marital status, ethnicity, dementia, serious/severe mental illness (SMI), anxiety, diabetes, circulation system diseases, exposure, dementia × exposure, SMI × exposure, and diabetes × exposure. Only the results of the interactions are shown.


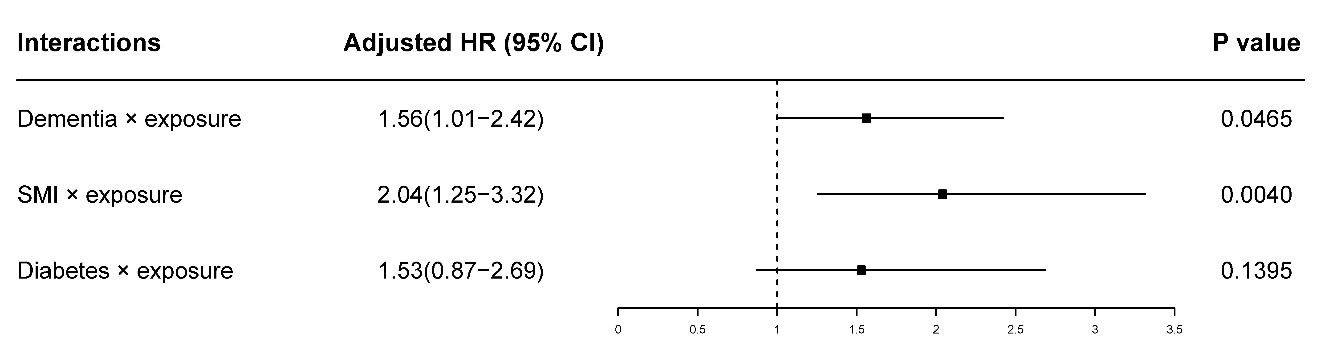


**Supplementary Figure2: Hazard ratios (HR) for excess death during lockdown, sensitivity analysis by considering possible seasonal effect.** Data are fitted by the Cox model. A binary variable (exposed v. unexposed) was treated as a time-dependent variable and as a stratification factor in the Cox model. Risk factors’ extra effects on death during lockdown were tested via the interactions between risk factors and exposure. After model selection based on Akaike’s Information Criterion (AIC), the final predictive factors included in the Cox model were age, gender, marital status, ethnicity, dementia, serious/severe mental illness (SMI), anxiety, diabetes, circulation system diseases, exposure, dementia × exposure, SMI × exposure, and diabetes × exposure. Only the results of the interactions are shown.


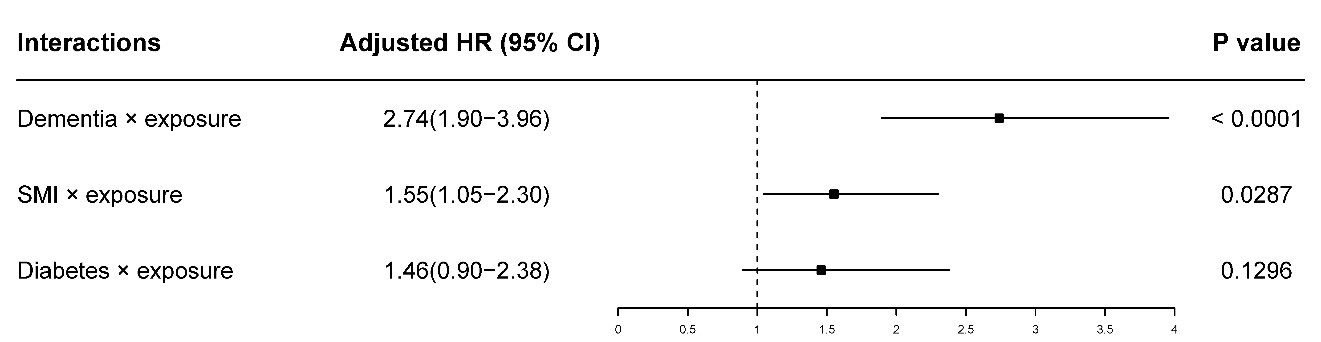


**Supplementary Figure 3: Hazard ratios (HR) for excess death during lockdown, sensitivity analysis by considering missing cases.** Data are fitted by the Cox model. A binary variable (exposed v. unexposed) was treated as a time-dependent variable and as a stratification factor in the Cox model. Risk factors’ extra effects on death during lockdown were tested via the interactions between risk factors and exposure. After model selection based on Akaike’s Information Criterion (AIC), the final predictive factors included in the Cox model were age, gender, dementia, serious/severe mental illness (SMI), anxiety, diabetes, circulation system diseases, exposure, dementia × exposure, SMI × exposure, and diabetes × exposure. Only the results of the interactions are shown.


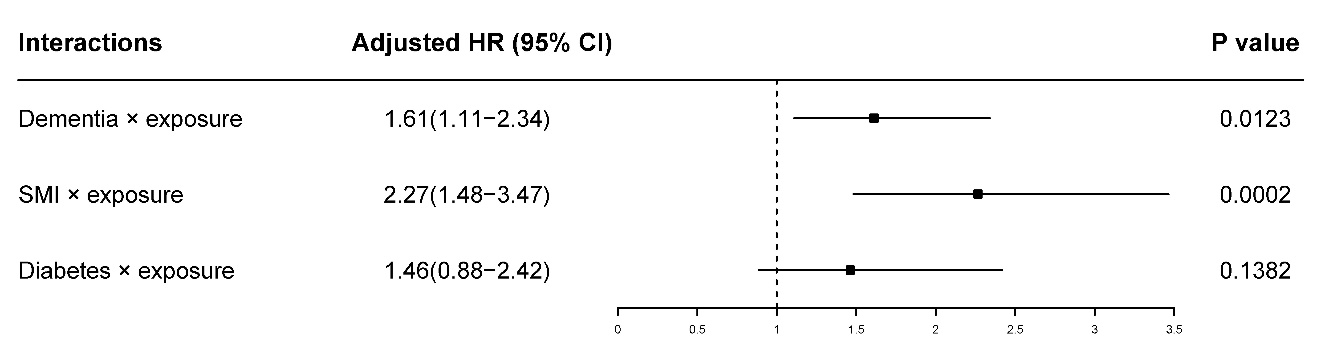

Supplement: Supplementary file 1 — Supporting Information S1 [file GPS-36-1899-s001.docx]
